# Monkeypox Virus in Nigeria: Infection Biology, Epidemiology, and Evolution

**DOI:** 10.3390/v12111257

**Published:** 2020-11-05

**Authors:** Emmanuel Alakunle, Ugo Moens, Godwin Nchinda, Malachy Ifeanyi Okeke

**Affiliations:** 1 Department of Natural and Environmental Sciences, Biomedical Science Concentration, School of Arts and Sciences, American University of Nigeria, 98 Lamido Zubairu Way, PMB 2250 Yola, Nigeria; emmanuel.alakunle@aun.edu.ng; 2 Molecular Inflammation Research Group, Institute of Medical Biology, University i Tromsø (UIT)—The Arctic University of Norway, N-9037 Tromsø, Norway; ugo.moens@uit.no; 3 Laboratory of Vaccinology and Immunology, The Chantal Biya International Reference Center for Research on the Prevention and Management HIV/AIDS (CIRCB), P.O Box 3077 Yaoundé-Messa, Cameroon; nsehleseh@gmail.com; 4 Department of Pharmaceutical Microbiology & Biotechnology, Faculty of Pharmaceutical Sciences, Nnamdi Azikiwe University, P.O Box 420110 Awka, Nigeria

**Keywords:** *Poxviridae*, orthopoxviruses, monkeypox viruses, epidemiology, Nigeria, signaling, phylogeny, gene loss, recombination, antiviral drugs

## Abstract

Monkeypox is a zoonotic disease caused by monkeypox virus (MPXV), which is a member of orthopoxvirus genus. The reemergence of MPXV in 2017 (at Bayelsa state) after 39 years of no reported case in Nigeria, and the export of travelers’ monkeypox (MPX) from Nigeria to other parts of the world, in 2018 and 2019, respectively, have raised concern that MPXV may have emerged to occupy the ecological and immunological niche vacated by smallpox virus. This review X-rays the current state of knowledge pertaining the infection biology, epidemiology, and evolution of MPXV in Nigeria and worldwide, especially with regard to the human, cellular, and viral factors that modulate the virus transmission dynamics, infection, and its maintenance in nature. This paper also elucidates the role of recombination, gene loss and gene gain in MPXV evolution, chronicles the role of signaling in MPXV infection, and reviews the current therapeutic options available for the treatment and prevention of MPX. Additionally, genome-wide phylogenetic analysis was undertaken, and we show that MPXV isolates from recent 2017 outbreak in Nigeria were monophyletic with the isolate exported to Israel from Nigeria but do not share the most recent common ancestor with isolates obtained from earlier outbreaks, in 1971 and 1978, respectively. Finally, the review highlighted gaps in knowledge particularly the non-identification of a definitive reservoir host animal for MPXV and proposed future research endeavors to address the unresolved questions.

## 1. Introduction

### 1.1. Introduction to Family Poxviridae

Poxviruses belong to family *Poxviridae*, a large and diverse family of double-stranded DNA viruses that multiplies in the cytoplasm of infected cells [1,2]. The poxviruses are known to have brick-shaped or oval structures measuring 200–400 nm when viewed with electron microscopy [3]. The wide host range of poxvirus, as well as their successful evolution, is partly due to their manipulation and modulation of host immune responses. The poxviruses are also called ancient viruses because they have been found in insects, reptiles, birds, and mammals. It is believed that these viruses, before the divergence of invertebrates and vertebrates, form visible “pox” [1,4,5,6].

The family *Poxviridae* is subdivided (based on their animal hosts) into two subfamilies, namely *Chordopoxvirinae* and *Entomopoxvirinae*. The former subfamily is known to infect vertebrates, and it is differentiated into 18 genera, including *Avipoxvirus*, *Capripoxvirus*, *Cervidpoxvirus*, *Leporipoxvirus*, *Molluscipoxvirus*, *Orthopoxvirus*, *Parapoxvirus*, *Suipoxvirus*, and *Yatapoxvirus*, while the latter subfamily is known to infect invertebrates, and it is grouped into four genera (*Alphaentomopoxvirus*, *Betaentomopoxvirus*, *Deltaentomopoxvirus,* and *Gammaentomopoxvirus*) [7]. The subfamilies of *Poxviridae* were each divided into its genera based on the shared antigenic similarity, induction of immunological cross protection, and phylogenetic grouping [1,4].

Although the infection biology and epidemiology of MPXV have been widely studied and published, there is still paucity of data and published results on the occurrence, distribution and virus transmission dynamics in Nigeria. Thus, the objective of this paper is to review the current state of knowledge concerning the infection biology, epidemiology, and evolution of MPXV in Nigeria. In addition, this review also chronicles and explores the knowledge gaps as they pertain to MPXV reservoir hosts and the ecological dynamics that modulate the maintenance of the virus in nature, as well as their spillover into human populations and subsequent human-to-human transmission.

### 1.2. History of Monkeypox

Monkeypox virus was first reported in 1959 as an outbreak of a pox-like disease in monkeys kept at a research institute in Copenhagen, Denmark [8]. The first human MPXV case in medical history was recognized when, on 1 September 1970, a nine-month-old child was admitted to the Basankusu Hospital in the Democratic republic of Congo (at that time, known as the Republic of the Congo). The boy had a smallpox-like disease from which MPXV-like virus was isolated [8,9,10,11]. Six cases of human MPXV were described in Liberia, Nigeria, and Sierra Leone between October 1970 and May 1971. The first index MPXV case in Nigeria was recorded in 1971, and 10 MPXV cases were reported between 1971 and 1978 [12]. Since then, several thousand human cases of monkeypox have been confirmed in 15 different countries, with 11 of them in African countries. Monkeypox was imported to the United Kingdom, the USA, Israel, and Singapore [13].

### 1.3. Monkeypox Virus: Morphology, Genome Organization, and Morphogenesis

Having the same morphological characteristics as other orthopoxviruses the morphology of MPXV reveals that virions are ovoid or brick-shaped particles which are enclosed by geometrically corrugated lipoprotein outer membrane. MPXV size ranges are known to be 200 by 250 nm [14,15]. Membrane bond as well as densely packed core containing enzymes, a double-stranded DNA genome, and transcription factors are protected by the outer membrane. Due to an electron microscopy fixation artifact, the core is being described as biconcave, and it has lateral body on each side [4,16,17].

The MPXV genome consists of a linear double-stranded DNA (≈197 kb) [18] covalently joined at its ends by palindromic hairpins, and the inverted terminal repeats (ITRs) are made up of hairpin loop, tandem repeats, and some open reading frames (ORF). Although MPXV is a DNA virus, its entire life cycle occurs in the cytoplasm of infected cells. All the proteins required for viral DNA replication, transcription, virion assembly, and egress are encoded by the MPXV genome. The genes encoding for housekeeping functions are highly conserved among OPVs and are present in the central region of the genome while those that encode for the virus–host interactions are less conserved and are located in the termini region [1,15,16,17,19,20,21,22,23]. In VACV (and most in likely MPXV) intracellular mature virus (IMV) and extracellular-enveloped virus (EEV) are two forms of infectious virions produced in poxvirus-infected cells. IMV is released on cell lysis, while EEV is released from cells via interaction with actin tails, and this is said to be the cause of rapid long distance spread of the virus within the infected host. Although the aforementioned features are for VACV, it is likely that these features are common to all OPVs [22]. However, cell-associated virions (CEVs) are formed following the microtubule-mediated transport of intracellular enveloped virus (IEV) to the cell periphery, in which the outer membrane of IEV fuses with the plasma membrane and remains attached to the cell surface. CEVs are mostly responsible for cell-to-cell spread [24]. IEV is formed when IMV is wrapped by a double membrane derived from early endosomal component [17] or the trans-Golgi network (TGN) [25]. However apart from IEV exocytosis, an alternative route for the formation of EEV is by the budding of IMV through the plasma membrane [26]. In the prototype VACV, virion morphogenesis can be defective resulting in non-infectious dense particles (DPs) [26,27], but this has not yet been reported for MPXV. In addition, unlike some strains of CPXV in which IMVs are occluded within A-type inclusions (ATI) [28,29], MPXV do not form ATIs or sequester IMVs into ATIs because of truncation in the *ATIP* gene [30].

## 2. Monkeypox Virus Infection Biology, Diagnosis and Treatment

### 2.1. Animal Models

In choosing an ideal animal model for a study of human disease, there are characteristics that the animals must possess in order to get a desirable result. The animal models must have similar mode(s) of transmission as in human cases, similar disease course, morbidity, and mortality as that of human cases, utilization of infection route identical to the natural transmission of the pathogen, and potentiality of obtaining disease with the same infectious rate as that of causing disease in humans [31].

Experimental infection of Guinea pigs and Golden Hamsters (being challenged with MPXV via intracardial, intranasal, oral, and route inoculation) showed that these animals were resistant to MPXV with the former (Guinea pigs) having no observable symptoms of the disease except edema at the inoculation site (foot pad), and the later (Golden Hamsters), in spite of large dosages of virus, showed no apparent signs of disease. Adult rabbits have no observable signs of the disease following oral inoculation with the same strain of MPXV, but acute illness, as well as, generalized rash was observed when the route of infection was intravenous [31,32].

When adult ground squirrels (*Spermophilus tridecemlineatus*) were challenged with 10^5.1^ pfu (plaque-forming unit) of the West African MPXV via intranasal or intraperitoneal inoculation, anorexia and lethargy (as symptoms) were shown within four to five days of infection, and no other noticeable symptoms. However, when challenged with the Congo Basin MPXV, the animals were found to have more rapid and uniform severe respiratory distress from the onset, and the animals died earlier than the West African MPXV challenged animals. A hundred percent and sixty percent mortality were recorded when Prairie dogs were exposed to 10^5.1^ pfu of West African MPXV via intranasal or intraperitoneal route of infection, respectively. According to Huston et al., generalized rash was observed in asymptomatic black-tailed prairie dogs (*Cynomys ludovicianus*) after 9–12 days of exposition to 10^4.5^ pfu of both Congo Basin or West African MPXV via an intranasal and scarification routes. The notable symptoms observed in the animals included lethargy, inappetence, nasal discharge, respiratory distress, morbidity, and mortality. Lesser morbidity and mortality rate were observed for the West African MPXV, and LD_50_ (median lethal dose) was found to be hundred-times higher for the West African clade compared to Congo Basin clade (1.29 × 10^5^ and 5.9 × 10^3^, respectively) in the prairie dog MPXV model via intranasal route [31,33,34].

Hatch et al. tested the efficacy of the smallpox vaccines Imvamune (a.k.a JYNNEOS^TM^) and ACAM2000^TM^ against 10^5^ pfu aerosolized monkeypox virus in *Cynomolgus macaques* (*Macaca fascularis*) via intranasal route. Besides the red patches on all MPXV challenged animals at the vaccination site, declination of body weight was also observed in most animals especially in those mock vaccinated with TBS (Tris-buffered saline) with 10–18% loss in weight, and there was a consistent increment in body weight of all surviving animals in the vaccination groups from day 14 post-challenged [35].

African dormice (*Graphiurus kelleni*), previously treated with Dryvax vaccine, were protected from mortality following challenge with 2 × 10^4^ PFU of MPXV-ZAI-79, and the unvaccinated group of dormice experienced uniform mortality [36]. The efficacy of the nucleotide analogue Cidofovir, via intranasal route, was tested in some African dormice, which were challenged with Congo Basin clade virus MPXV-ZAI-79. Nineteen percent mortality rate was observed in animals treated with Cidofovir while vehicle or placebo treated animals were susceptible to the disease. The antiviral drug Tecovirimat (ST246) was administered to prairie dogs, via the oral route of infection, after they had been exposed to 65 × LD_50_ of Congo Basin clade virus ROC-2003-358, and generalized rash coupled with 10–24% decrease of animals’ body weight was observed. There was deterioration of the symptoms of the disease, as well as an increase in viral titers, in untreated groups of animals. However, all animals (at rash onset) in the treatment group survived. Thus, ST246 was established to provide potent antiviral activity against MPXV infection [31].

Out of the 38 inbreed strains of mice (32 classical inbreed strains and six wild-derived strains) screened for susceptibility to MPXV, only three (CAST/EiJ, PERA/EiJ, and MOLF/EiJ) of the wild derived strains were highly susceptible to MPXV, and all the classical inbreed strains were highly resistant to intranasal MPXV infection. CAST/EiJ (abbreviated as CAST) is the most sensitive (of the three susceptible mouse strains) to MPXV with LD_50_ of 680 and 14 infectious units via intranasal and intraperitoneal infections [37,38,39,40]. CAST was also used as a model for testing efficacy of smallpox vaccines and antivirals (Dryvax and Cidofovir) against MPXV. Although both therapeutics gave protection to MPXV-infected CAST mice, Dryvax was more effective than Cidofovir [39].

### 2.2. Transmission

The two possible means of MPXV transmission are animals–human transmission and human–human transmission. Respiratory droplets and contact with body fluids, contaminated patient’s environment or items, skin lesion of an infected person have been found to be associated with inter-human transmission. Congo Basin clade (Central Africa clade) is reported to be more virulent than West Africa clade and thereby contributes more to inter-human transmission [41,42,43,44,45,46]. Animal-to-human transmission, which is also known as zoonotic transmission, occurs via direct contact with any of the aforementioned natural viral hosts or consumption of these hosts. In addition, zoonotic transmission could occur by direct contact with the blood, body fluids, and inoculation from mucocutaneous lesions of an infected animal [41,47,48,49,50]. Nosocomial transmission has been reported for CB and WA clades of MPXV [51,52,53] while sexual transmission has been speculated for infected individuals with groin and genital lesions [54]. At present human-to-animal transmission has not been reported. Human-to-human transmission, secondary attack rates (SARS), and serial transmission events is much higher with the CB clade compared to the WA clade [55]. The reproduction number R0 for the CB clade is estimated to be in the range of 0.6–1.0 [56,57]. The R0 has not be estimated for the WA clade of MPXVs, but it is presumed to be lower than that of the CB clade. The upper limit R0 of 1.0 in the CB clade indicates that the viruses will not only sustain human-to-human transmission but may persist in human population. Presumably, if as expected the R0 of the WA clade is much lower than what was estimated for the CB clade, then sustained human–human transmission and persistence in human population are highly unlikely and outbreaks will be largely due to spillover events from zoonotic hosts.

### 2.3. Diagnosis

#### 2.3.1. Genetic Methods

This involves using PCR or real-time PCR (RT-PCR), and it is advisable that this test is carried out in a Biosafety level-three facility [58]. Routine detection of MPXV DNA from clinical and veterinary specimens, as well as from MPXV-infected cell cultures, is accomplished by RT-PCR targeting conserved regions of extracellular-envelope protein gene (*B6R*) [59], DNA polymerase gene, *E9L* [60]. DNA dependent RNA polymerase subunit 18, *rpo18* [61], and *F3L* gene [62]. Restriction length fragment polymorphism (RFLP) of PCR-amplified genes or gene fragments are also used in detection of MPXV DNA [63,64,65] but RFLP is time consuming and does require virus culture. RFLP of PCR products which also require enzyme digestion followed by gel electrophoresis and thus may not be the appropriate method in clinical setting where rapidity, sensitivity, and the specificity of the method is paramount. Whole-genome sequencing, using next-generation sequencing (NGS) technologies, remains the gold standard for characterization of MPXV and other OPVs [66,67,68,69], but the technology is expensive, and downstream processing of sequencing data requires enormous computation power. Thus, NGS may not be the appropriate method for characterization especially in the resource poor countries of sub-Sahara Africa. Although RT-PCR remain the method of choice for routine diagnosis of MPXV, it has to be complemented by field genome sequencing technology, like Oxford Nanopore MinION, in order to provide real-time virus genome data, which are indispensable for evidence-based epidemiological interventions. MinION field sequencing was successfully used for genome surveillance of Ebola outbreak in resource limited settings of West Africa [70].

#### 2.3.2. Phenotypic Methods

Based on the clinical diagnoses, the incubation period of MPXV is within 4–21 days, and it is usually followed by a prodromal illness with several signs including lymph node enlargement, headache, fever, back pain, myalgia, intense asthenia, severe headache, pharyngitis, drenching sweats, and malaise. The exanthema phase, following the prodromal phase, comes with vesiculopustular rashes that appear within 1–10 days of development spreading all over the body starting with the face. The lesions in MPXV patients appear monomorphic, pea-sized and hard which is similar with that of smallpox. Crop-like appearance of MPXV lesion and its non-strong centrifugal spread make it different from smallpox. Presence of lymphadenopathy in MPXV is the notable clinical feature that differentiates it from smallpox [6,42,43,58,71,72]. Presumptive identification of MPX based on clinical symptoms is important for identification of suspected cases during surveillance but clinical case definition for MPX in the absence of laboratory confirmation have been shown in a cohort of 645 individuals to have high sensitivity (93–98%) and low specificity (9% to 26%) [55,73].

#### 2.3.3. Immunological Methods

This includes using enzyme-linked immunosorbent assay (ELISA) for IgG and IgM antibodies detection and immunohistochemistry for viral antigen detection. Immunochemistry analysis can be used to differentiate between poxvirus infection and herpes virus, using polyclonal or monoclonal antibodies against all OPVs. It has been established that antiviral antibody, as well as T-cell responses, increases around the time of disease onset. However, IgM and IgG are detected in serum about five days and more than eight days after the onset of rash, respectively. Indirect MPXV diagnosis may arise if IgM and IgG antibodies are present in an unvaccinated individual with a history of rash and severe illness. However, none of these methods is specific for MPX [43,44,46,58,71,74] and can indicate the presence of other OPV species. IgM can be used to assess MPX infection in an individual with prior history of smallpox vaccination [75]. Positive IgM capture ELISA is indicative of recent exposure to OPV (likely MPXV in endemic areas) in both naïve and vaccinated individuals whereas a positive IgG capture ELISA suggests that the individual has been previously exposed to OPV either through vaccination or natural infection [76,77]. Consequently, the presence of both IgM and IgG in a sample is a strong evidence for recent exposure to OPV in individuals that has been previously vaccinated or exposed to natural infection. Thus, the presence of IgM in individuals previously vaccinated against smallpox in regions of MPX endemicity is indicative of a recent exposure to MPXV.

#### 2.3.4. Electron Microscopy

MPXV under electron microscope appears intracytoplasmic brick-shaped with lateral bodies and a central core measuring about 200–300 nm. Although this method is not definitive diagnosis because OPV species cannot be differentiated morphologically, but it gives a clue that the virus belongs to *Poxviridae* family [6,43,71,78].

### 2.4. Virus–Host Interaction

#### 2.4.1. Host and Tissue Tropism

Horizontal gene transfer (HGT) has been an obvious explanation for accumulation of immune evasion genes (tumor necrosis factor (TNF) receptor, interferon (IFN)-γ receptor, MHC class 1, interleukins among many others) among poxviruses, and the ability of poxviruses to become host-restricted could be ascribed to the coevolution of the viruses and their hosts over many years [1].

McFadden highlighted three levels of viral tropism (cellular tropism, host or organism tropism, and tissue tropism) [79]. Tissue and host tropism affect, to a large extent, the distribution and propagation of the virus in an infected hosts and between hosts, respectively [79]. Members of the genus OPV are believed to have different spectrum of host tropisms, and such variability may enhance host–virus interface, reduction in pathogenesis, and different immune evasion molecules [1,80,81,82,83]. Although the reservoir host of MPXV has not been definitively identified, many mammalian species have been naturally infected with MPXV (Table 1). Thus, MPXV is presumed to have a wide host range. Huston et al. detected high levels of viral DNA and viable virions in a wide range of tissues in animals that died following challenge with Congo Basin MPXV [80], and this may suggest a wide tissue tropism.

Immunohistochemistry and histopathology tests (using mice with severe combined immunodeficiency) conducted by Osorio et al. have revealed that MPXV antigen was detected in the ovarian tissue, brain tissue, heart tissue, kidney tissue, liver tissue, pancreatic tissue, and lung tissue; however, the viral titers were higher in ovarian tissues than other tissues, and therefore ovarian tissues proved to be highly susceptible to MPXV [110]. The histopathological studies conducted on cynomolgus monkeys (*Macca fasicularis*) by Zaucha et al. showed that lymphoid tissues are good tropism for MPXV, and the viral antigen was also detected in the salivary epithelium, follicular, sebaceous tissue of the lip among many other tissues [111]. The presence of MPXV DNA in multiple tissues and virus isolation in different animals (like dormice, praire dogs, giant pouched rats, rope squirrels and others) highlight the difficulty in targeting a particular tissue as a site of infection [99,112].

#### 2.4.2. Signaling in Orthopoxvirus Infection

Viruses are obligate intracellular parasite [113]. Replication of viruses inside their host cells serves as means of viral propagation, and this possible because viruses are host-dependent for their survival. The adaptability of viruses to their hosts involves manipulation of host cell signaling. Signaling in any viral infection is very crucial as it serves as portal of entry to viral genomes or proteins as the case maybe, and it usually determines the outcome of an infection [114]. The two signaling networks most targeted by viruses are cell growth and immunoregulation [115]. Inhibition or retardation of apoptosis, suppression of antiviral host defense, and exploitation of host cell machinery are well-known features of OPVs [116].

Of the three classical mitogen-activated protein kinase (MAPK) pathways (MEK-ERK, Jun amino-terminal kinases or JNK and p38/SAPK or stress-activated protein kinase) [117], the MEK (MAPK/ERK kinase)-ERK (extracellular signal regulated kinase) cascade is induced by many DNA and RNA viruses to create an optimal intracellular environment for their replication cycle. Extracellular signals which induce cellular proliferation, differentiation, and survival are transmitted primarily by the MAPK-ERK pathway (the central regulator of cellular response to environmental stimuli). Alteration of MAPK-ERK signaling by DNA viruses dysregulates the cell cycle, promotes viral internalization, regulates viral replication, and prevents host-cell death. The MAPK-ERK pathway is activated upon initial VACV infection so as to promote the expression of viral proteins like O1 which is needed for viral replication. The MAPK-ERK activation is sustained by the expression of O1 [113].

Deacetylating histones is a way implored by histone deacetylases (HDACs) to repress gene expression which in return strengthens histone DNA interactions and chromatin condensation. The 18 HDACs found in human are characterized into four groups, namely class I (HDACs 1,2,3, and 8), class III (sirtuins 1–7), class IV (HDAC 11), and class II, which is subdivided into class IIa (HDACs 4,5,7, and 9) and class IIb (HDACs 6 and 10). Lu et al. used the osteosarcoma U2OS cell line to determine the effect of HDAC4 on the replication and spread of VACV and HSV-1, and found that HDAC4 is not only required for type I IFN signaling, but it also restricts the replication and spread of the viruses due to the overexpression. Similarly, the enhancement of VACV replication and spread was observed due to the loss of HDAC4. Protein C6 and the proteasome are responsible for HDAC4 degradation during VACV infection [118].

There are large number of members in the heat shock proteins (HSPs) family including DNAJ (HSP40), HSPD, HSPA (HSP70), HSPC (HSP90), HSPH, and HSPB (small HSPs). These HSPs have multiple isoforms and act as chaperons that help in protein folding [119]. Heat shock factor 1 (HSF1), the regulator of cytoprotective heat shock response, is necessary for OPV infection. Filone et al. discovered that the VACV early, intermediate, and late gene expressions were reduced following the depletion of HSF1, and this suggests that HSF1 is essential for VACV lifecycle. An increase in the phosphorylated form of HSF1 following the VACV infection suggests the activation of HSF1, and translocation of phosphorylated HSF1 to the nucleus indicates that VACV activates HSF1 in a similar manner to heat shock. Furthermore, the VACV infection reported to reduce following the activation or reduction of heat shock protein activity by HSF1 inhibitors (triptolide, KNK437, quercetin, KRIBB11, KRIBB3, myricetin, and gentespib (STA-9090)) [120,121].

Shchelkunov noted that the cell antiviral signaling and inflammatory responses are activated by the viral infections, and the nuclear factor kappa B (NF-κB) is crucial for many responses like cell proliferation, apoptosis, inflammation, and immune responses. He further stated that cellular protein kinase phosphorylates IκBα kinase (IKK) in order to phosphorylates IκBα at the serine residue at positions 32 and 36, respectively, and the ability of OPVs to block the function of STAT (signal transducer and activator of transcription) proteins (which serves as antiviral responses) has become the common mechanism or strategy for viral immune evasion. MPXV has eight ankyrin (ANK) genes, and ANK controls the activation of NF-κB. VACV A46R, CPXV A49R, MPXV A47R, and VARV A52R are Bcl-2-like proteins that inhibit activation of NF-κB and IRF3 by interacting with MyD88, TIRAP and TRIF, and TRAM. MPXV B13R interacts with IKK so as to inhibit the activation of NF-κB [122,123].

Wiskott-Aldrich syndrome protein (WASP) and elevated ATP level are essential for VACV infection. Nicotinamide adenine dinucleotide dehydrogenase 4 (ND4) and cyclooxygenase-2 (COX2) are mitochondrial proteins which generate ATP in the electron transport chain and are said to be increased by VACV. Liem and Liu stated that the components of the eukaryotic translation initiation complex eIF4F are recruited to viral factories during VACV infection, and these components are m7G-cap-binding subunit (eIF4E), a small RNA helicase (eIF4A), and a large scaffold protein (eIF4G). The eIF4G with its associated proteins, during VACV infection, are brought into close propinquity with viral factories by the single-stranded DNA (ssDNA) binding phosphoprotein I3. The MAP kinase-activated protein kinase Mnk1, which has a direct association with eIF4G, regulates eIF4E activity and promotes eIF4E phosphorylation. ERK1/2MAP kinases and p38/SAPK regulate Mnk1 activity. The activation of ERK and p38, which occurs during VACV infection, is associated with eIF4E activation. Deletion or inhibition of Mnk1 hinders VACV infection severely. Therefore, VACV boost the viral protein synthesis by manipulating Mnk1 activity in addition to sequestering of translation initiation complex [120].

Members of integrins family are cell surface receptors with 18 different α and eight different β subunits. Multiple α subunits which include α1-11 and αV are associated with integrins β1. Integrins β1, via interaction with the extracellular matrices, regulates multiple intracellular kinase activation pathways [124]. During the experiment carried out by Izmailyan et al., using HeLa cells and mouse cells, respectively, VACV entry was reduced upon knockdown and knockout of integrins β1. The activation of phosphophatidylinositol-3-kinase (PI3K)/Akt, which leads to virus endocytosis in HeLa cells, was observed to occur as a result of binding of Vaccinia MV to integrins β1. ERK, protein kinase C, and p21-activated kinase 1 are said to be activated by VACV [124], but the biological relevance for VACV replication is not known.

#### 2.4.3. Host Immune Responses to MPXV

There are many mechanisms or strategies developed by PXVs to evade or worsen host immune response to infection [125]. More studies on the pathology and pathogenesis of MPXV have been done, but knowledge of innate and adaptive immune response of MPXV infection is not well understood due to insufficient data. Natural killer (NK) cells, a major component of innate immunity, directly kill virus-infected cells by secretion of cytokines in order to modulate functions of other cell types like T-cells and dendritic cells. The interaction between activating or inhibitory receptors on NK cells and their ligands like major histocompatibility complex 1 (MHC-1) molecules initiates activation or inhibition of NK cells. Secretion of granules (containing perforin and granzymes) and cell–cell interactions mediate the killing effect of NK cells. Inflammatory responses in inflamed tissues are mediated by IFN-γ and TNF-α that were secreted at early stages of infection by NK cells, and these cytokines are also involved in coordinating the dendritic cells to induce T-helper type 1 (Th1) cell polarization [126].

Earl et al. observed that CAST was highly susceptible to MPXV, and inadequate or insufficient interferon-γ response in the lung is correlated with the vulnerability of CAST mice following intranasal infection [37,38,40]. Similarly, susceptibility of CAST mice to MPXV is due to low numbers of NK cells. CAST mouse NK cells, which were purified and expanded in vitro with IL-15, were protected against MPXV [40].

According to Song et al., MPXV infection is responsible for changes in the number of lymphocytes including NK cells in NHPs, and lymphadenopathy, as well as lymphoid depletion, in MPXV-challenged NHPs, is a result of MPXV infection [127]. At the end of an experiment conducted by Townsend et al., using Prairie dogs as models, there was a significant increase in the following: the number of all NK subsets at day seven postinoculation, CD16− CD56−, CD16+, CD56+, and CD16+CD56+ NK cells. The number, as well as composition, of NK cells in the lymph nodes during MPXV infection was observed to increase by 4.6%, and the observations made on the expression of chemokine receptors (CXCR3, CCR5, CCR6, and CCR7) on each NK cell subset suggest that, following the MPXV challenge, the receptors expression was delayed or reduced [126].

Studies have shown that MPXV disseminate poorly by inter-human contact [128]. and VARV and MPXV, as opposed to VACV, make use of cell-associated viremia to spread through their infected hosts [110,128,129,130,131]. Although the mechanisms involved in immune evasion of poxviruses against antiviral cytokines, chemokines, and antigen presentation are not well understood, interference with intracellular transport of MHC class I by CPXV also correlate the mechanisms used by CPXV in evading antiviral CD8+ T-cell responses [128].

Hammarlund et al. expected MPXV to have similar mechanisms of immune evasion as CPXV because MPXV encodes a close homologue of CPXV203 which retains MHC class I in the endoplasmic reticulum. However, the mode of evasion mechanism used by MPXV inhibited the activation of CD4+ and CD8+ T cell after cognate interactions with MPXV-infected cells, and this protects the viral reservoir from immune surveillance. Poor recognition of MPXV-infected monocytes by antiviral CD4+ and CD8+ indicated that MPXV did not trigger inflammatory cytokine production (IFNγ or TNFα) via virus-specific T cells, but the virus is efficient in infecting primary human monocytes [128].

Antiviral T cell responses increased greatly after only VARV infection, but following co-infection of MPXV and VARV, T-cell-mediated cytokine responses reduced by 95% and dropped by 80% when a low dose of MPXV was added (VARV:MPXV ratio of 10:1). This result indicated that T cell activated by VARV was inhibited by MPXV, and the immunomodulatory protein that is absent from VARV is encoded by MPXV. After peripheral blood mononuclear cells had been infected or uninfected, in the presence or absence of cytosine arabinoside (AraC) to block late gene expression, with VARV or MPXV, an early gene product was observed because inhibition of anti-CD3 responses in the presence of AraC was detected. This indicates that an early gene was responsible for immunosuppression of T cells (CD4+ and CD8+) by MPXV [128]. The identify of this gene remains unknown.

In Nigeria, there is no report on T cell and antibody responses to human MPXV infections. There are also no data or reports on immune responses following experimental infections in animal models. Yinka-Ogunleye et al. reported that IgM ELISA was carried out on the cases during the 2017–2018 MPX outbreak but the results were not published. However, smallpox vaccination ceased in Nigeria since 1970. Thus, those 50 years or below were not vaccinated against smallpox and presumably are not protected against OPV infections including MPXV. As at 2019, about 90.2% of the 200 million Nigerians are under 50 years, of which 62.9% are 24 years or younger [132,133,134]. The absence of cross-protection elicited by smallpox vaccine, the probable waning of herd immunity and immunosuppression as a result of HIV/AIDS have in tandem resulted in an immunologically naive population that is very susceptible to MPXV infection. This may in part explain the recent re-emergence of MPXV in Nigeria.

### 2.5. Treatment

#### 2.5.1. Vaccination

Studies have shown that vaccination against smallpox provides cross-protection against other OPV species including MPXV. According to the available data, about 90% of the identified cases are naive to OPV infection of which many of them were born after the cessation of smallpox eradication program [46]. Individuals who had previously been vaccinated against smallpox were identified to have 85% protection against MPXV [43,46,74,78]. The smallpox vaccine (ACAM2000^TM^) recommended by the Center of Disease Control and Prevention (CDC), during the 2003 USA MPXV endemic, proved to reduce the symptoms and did not prevent disease [46]. Therefore, this vaccination is neither available to the public nor used in MPXV endemic areas due to some hiccups like unknown effects of the vaccine in immunosuppressed individuals and safety of the vaccine containing live vaccinia virus. IMVAMUNE, a replication deficient, attenuated third generation modified vaccinia Ankara (MVA) vaccine, has also been licensed by Food and Drug Administration (FDA) and the European Medicine Agency (EMA) for the prevention of smallpox and monkeypox for adults (18 years or older) determined to be at high risk of infection to VARV and MPXV [135,136]. Unlike ACAM2000, IMVAMUNE is not contradicted for use in individuals with immunodeficiencies such as AIDS and atopic dermatitis [135,136]. Currently, neither ACAM2000 nor IMVAMUNE have been approved for use in the general population. Consequently, whether or not these licensed smallpox vaccines will provide effective protection against MPX in MPXV endemic areas remain to be determined [43,46,50,71,74].

#### 2.5.2. Antivirals

A 4-trifluoromethylphenol derivative or Tecovirimat (ST-246 or TPOXX^®®^), having been approved by the FDA, has undergone clinical trial, using an animal model. The drug has proven to be effective in the infected animals by blocking the release of the intracellular virus from the cell. According to CDC report, the human clinical trial with Tecovirimat suggested that the drug was tolerable and safe, but there are not enough data on its effectiveness in treating human cases of MPX. Similarly, animal and in vitro studies, using cidofovir and Brincidofovir (CMX001 or hexadecyloxypropyl-cidofovir), proved to be effective. These two drugs inhibit the viral DNA polymerase [42,46,58,71,78,137,138,139], and the former is an acyclic nucleoside phosphate while the latter is a liquid conjugate of cidofovir [140]. The cellular cytotoxicity of TPOXX^®®^ in several cell lines, including human, was >50 µM, and interaction of TPOXX^®®^ with *F13L* gene product inhibits the production of extracellular viruses. Meanwhile the formation of protein complex, which catalyzes the envelopment of intracellular mature virus particles, requires phospholipase that is encoded by *F13L* gene product [140,141].

Although the Brincidofovir has higher cellular toxicity and better antiviral activity than cidofovir against VARV, MPXV, VACV, and CPXV in vitro, Brincidofovir has greater selective index due to its better efficacy which was at least 25-fold higher than cidofovir’s. Increased cellular uptake alongside with better conversion to the active form by intracellular enzymes are responsible for better efficacy seen in Brincidofovir. Cidofovir is a monophosphate nucleotide analog and incorporation of a second phosphate group by cellular kinases converts it into an inhibitor of the viral DNA polymerase, which is also tantamount to inhibition of viral DNA synthesis by Brincidofovir activity. The phosphorylated cidofovir is formed via the conversion by intracellular kinases of cidofovir which is released by cleavage after Brincidofovir has entered the cells through the endogenous liquid uptake pathways [140].

Aside from the aforementioned drugs, Delaune and Iseni had done justice in exploring or evaluating the possibility of other potential drugs with antiviral activity against poxviruses. Nucleoside analogue inhibitors (N-Methanocarbathymidine, 4′-thio derivative of idoxuridine, and KAY-2-41) were among the potential drugs that were tested due to their antiviral activity. The efficacy of *N*-methanocarbathymidine (N-MCT) was observed in a mouse model infected with VACV, Balb/c mice infected with CPXV Brighton, and against herpesviruses. The N-MCT triphosphate metabolite, whose formation is viral thymidine kinase-dependent, mediates the antiviral activity of the drug. In vitro studies revealed that 4′-thio derivative of idoxuridine (4′-thioIDU) showed efficacy against CPXV, as well as VACV and against viral strains which are resistant to cidofovir or Tecovirimat. The efficacy of KAY-2-41 OR 1′-Carbon-substituted 4′ thiothymidine derivative had been said to be greater than cidofovir but lower than Brincidofovir or Tecovirimat. It had been shown that KAY-2-41 provided protection against VACV, CPXV, and CMLV in vitro. Another potential drug tested against poxviruses was NIOCH-14 which is a derivative of tricyclodicarboxylic acid and a precursor of Tecovirimat. Even though the efficacy of NIOCH-14 was similar to Tecovirimat in in vitro studies against VARV, MPXV, and ECTV, but NIOCH-14 remains a relevant antiviral candidate for the future due to its potent antiviral activity against many OPVs, and its production is easier than Tecovirimat [140].

Baker et al., were able to test potential drugs against OPVs and grouped these drugs into five categories (*S*-adenosylmethione, Inosine monophosphate (IMP) dehydrogenase, DNA polymerase inhibitors, reverse transcriptase (RT) and protease inhibitors, and other compounds) based on their antiviral activities. Ribavirn and tiazofurin, which are IMP dehydrogenase inhibitors, proved to inhibit the replication of all the tested OPVs with VARV and MPXV being more sensitive than the other viruses. The protease inhibitors (saquinavir, ritonavir, and nelfinavir) and reverse transcriptase inhibitors (efavirenz, stavudine, and zidovudine) were inactive against OPVs while the two adenosine analogs (C-ca3-Ado and C3-Npc A) were shown to have protective effect against the tested OPVs by the viral replication, and these drugs are also *S*-adenosylhomocysteine (SAH) hydrolase inhibitors. Even though with a wide-range of antiviral activity of these SAH hydrolase inhibitors, they showed no detectable activity against CPXV in vitro. The DNA polymerase used by Baker et al. are the following: acyclovir, brovavir, lobucavir, cidofovir, adefovir (PMEA), adefovir dipivoxil (bis-POM-PMEA), and [(S)-9-(3-hydroxy-2-phosphonomethoxypropyl) adenime] or (HPMA), and they are all nucleoside analogs. The first three nucleoside analogs failed to be effective in inhibiting the OPVs replication, and this could be as a result of inability of the OPV thymidine kinase to recognize the drugs as a substrate for phosphorylation. Cidofovir and HPMA showed activity against PXVs by inhibiting the viral replication, using the same mechanisms, but adefovir and dipivoxil showed no significant activity against poxviruses. In addition, adenosine N1-oxide (ANO) showed significant activity against the OPVs by blocking the translation of viral mRNAs and thereby inhibiting viral replication [142]. There has not been any specific curative treatment for MPXV, it is only managed through supportive care and symptomatic treatment [143,144], but the problem with this type is that it could only cater for symptomatic individuals. Therefore, effective vaccination or antiviral drugs against MPXV are needed to prevent spread from asymptomatic individuals to other people.

## 3. Ecology and Epidemiology of Monkeypox Virus in Nigeria

### 3.1. Geographic Distribution of Human Monkeypox in Nigeria

**Figure 1:**
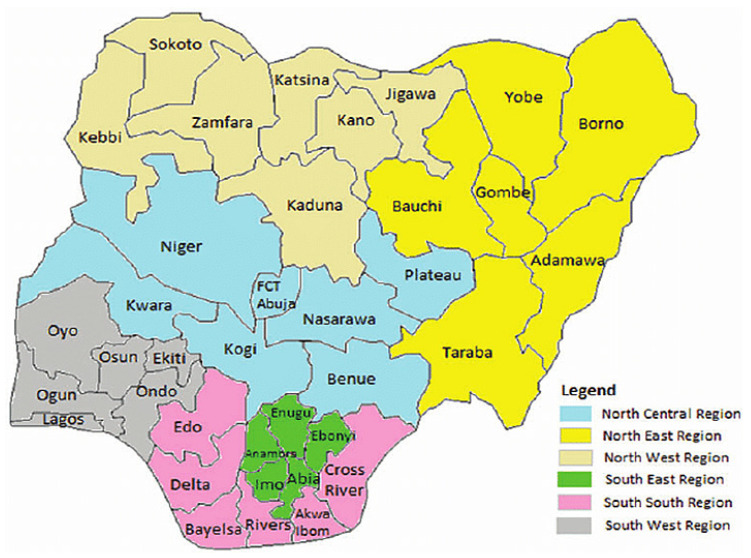
Map of Nigeria [148], reproduced with permission from the authors and Oxford University Press (Oxford, England).

Nigeria is a sovereign country located in West Africa with a population of over two hundred million [133,145]. The country is made up of 36 states and federal capital territory as illustrated in Figure 1. MPX has been reported in some regions or states in Nigeria. As at 27th October 2017, the Nigeria Center for Disease Control (NCDC) reported some cases to WHO, and these cases were confirmed in Abuja, Enugu, Bayelsa, and Akwa Ibom. The suspected cases were from Delta, Nassarawa, Niger, Rivers, Abuja, Lagos, Imo, Enugu, Ekiti, Akwa Ibom, Bayelsa, and Cross River. In January 2019, many suspected cases were reported across 26 states while confirmed cases were recorded in 17 states [41,42,52,58,144,146,147].

NCDC reported, as at September 2019, MPXV cases across nine states which include Oyo, Bayelsa, Lagos, Delta, Rivers, Enugu, Akwa Ibom, Anambra, and Cross River [43,47,149,150]. Meanwhile MPXV cases were reported in six additional states (Imo, F.C.T Abuja, Bauchi, Zamfara, Borno, and Plateau) as at December 2019, and this makes the total reported cases to 113 and confirmed cases to 45 between January and December 2019 [151]. Although there are not enough data to suggest that this disease is endemic in any region of Nigeria, some states in the Southern Nigeria (Southwest, South-South, and Southeast) happened to record outbreaks of the disease over the years.

### 3.2. Reservoir Host Species of Monkeypox Virus in Nigeria

Even though the natural reservoir of MPXV in Nigeria is yet to be known, isolation of MPXV from the Gambian pouched rat, tree squirrel, rope squirrel, and sooty mangabey monkey, among other primates, suggests that the reservoir species are African rodents [46,48,55,60,149,152]. However, there is a lack of data on potential reservoir species of MPXV in Nigeria. Currently, there are no published reports on the presence of MPXV in small mammals and rodents in Nigeria. The mangrove and tropical rainforest of Southern Nigeria where most of the recent monkeypox outbreaks have occurred is rich in different species of small mammals, rodents, and primates [153,154,155]. Serological, genetic, and virus isolation epidemiological surveys of rodents, small mammals, primates, and wildlife for MPXV should be conducted to identify the animal reservoir, and natural and incidental hosts of MPXVs in Nigeria. Future studies should also look at the possibility of MPXV not having natural reservoir(s) but circulates in a wide variety of natural and incidental animal host species.

### 3.3. Epidemiology of Monkeypox Virus in Nigeria

In Nigeria, the first human case of MPXV was recorded in a four-year old girl in 1971, and the second case was the mother of the four-year old girl. The affected individuals were residents of Ihie Umduru located in the present Abia state, and the mother was presumed to have gotten infected by her child. Similarly, the third case of MPXV in Nigeria occurred in 1978 in a thirty-five-year old man living in Omifunfun (Oyo state) [12,54,60,147]. Out of 10 reported cases between 1971 and 1978, only three cases were confirmed, and zero death was recorded [156,157].

**Figure 2:**
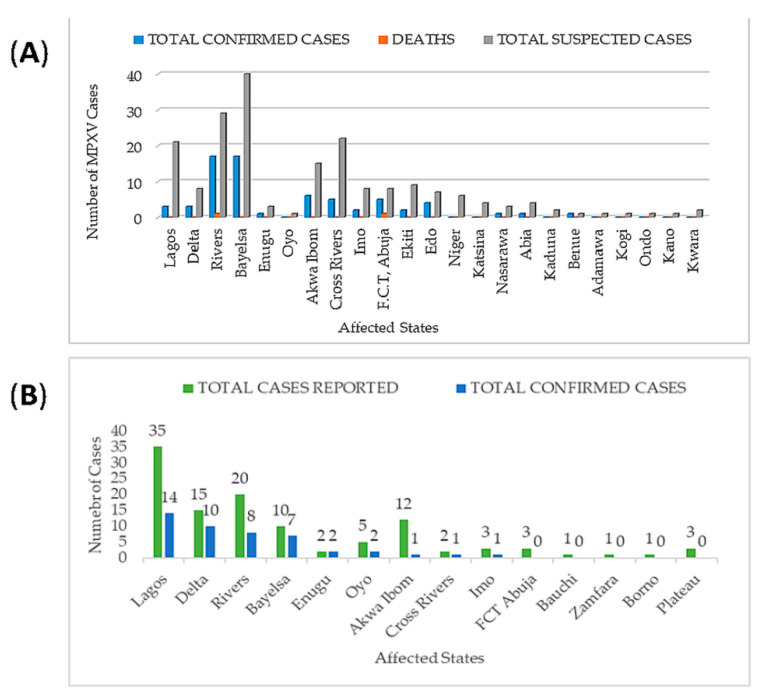
(**A**) Total MPXV cases across affected states in Nigeria, in 2017. (**B**) Human MPXV cases across Nigeria states, in 2019.

After almost 40 years of no reported MPXV cases in Nigeria, there was reemergence of MPXV in September 2017. The Niger Delta University Teaching Hospital (NDUTH) notified NCDC of a suspected MPXV case in an eleven-year old with an eleven-day history of fever, generalized rash, headache, malaise, and sore throat [52,54,55,74,147]. One hundred and ninety-seven suspected cases across 23 states in Nigeria had been reported since the onset of MPXV outbreak in September to December 2017 [157]. The highest numbers of suspected cases were recorded in Bayelsa, Rivers, Lagos, Cross River, and Akwa Ibom with 40, 29, 21, 22, and 15 cases, respectively (Figure 2A). A total of 68 confirmed cases was recorded in 2017, and two deaths reported were from two states. Only nine states out of 23 states with suspected cases had no confirmed cases while 14 states had between 1 and 20 confirmed cases [158].

As at 13 November 2018, 104 suspected cases had been recorded (by NCDC) in 19 states, 38 confirmed cases in twelve states, and one death in Imo state. Rivers, Cross River, Bayelsa, and Akwa Ibom states constitute 68% of suspected cases while Rivers, Bayelsa, Delta and Oyo states constitute 66% of confirmed cases [159]. Lagos, Rivers, and Delta states were at the vanguard of 2019 MPXV cases with 35, 20, and 15 suspected cases and 14, 10, and 8 confirmed cases, respectively (Figure 2B) [151].

**Figure 3:**
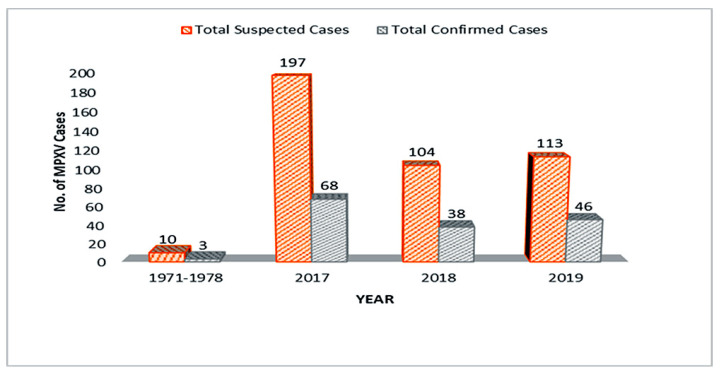
All human MPXV cases in Nigeria over the years.

By and large, Nigeria has a record of 424 MPXV suspected cases and 155 confirmed cases with the highest number of confirmed cases in 2017 outbreak (Figure 3). A declination in the number of confirmed and suspected cases in 2018 could suggest that Nigeria’s efforts in combating MPXV proved to be effective, but the 2019 data suggested otherwise (Figure 3). MPXV cases between 2017 and 2019 were reported in the 21-to-40-year age group (median age = 30) [52,159,160,161], with the male-to-female ratio of confirmed cases being 3:1 [151,158,159,160]. Although no MPXV cases have yet been recorded or reported in 2020, this could be due to many factors particularly the migration of resources and personnel from MPXV surveillance and response to more deadly viral diseases especially covid-19 and Lassa fever [162,163]. Virtually the whole resources of NCDC have been channeled to battling covid-19 pandemic and Lassa fever epidemics in 2020.

The reemergence of MPX in Nigeria after more than three decades of no reported cases maybe due to the fact that a large section of the Nigerian population is immunologically naïve to OPV infection as they did not receive smallpox vaccination which cross protects against monkey pox or the smallpox vaccine induced immunity have waned in vaccinated individuals [126,164,165,166]. Other factors for the re-emergence will include (i) increased encroachment of the wildlife habits of human and non-human primates by humans due to urbanization and hunting, (ii) increased trade in rodents and other species of wildlife fueled by the increased demand for and consumption of barbequed rodents/wildlife mammals (referred to as “bush meat”) in Nigeria, (iii) heavy rainfall and flooding that brought humans and MPXV-infected animal host close together, (iv) immunosuppression due to co-infection with HIV, and (v) a young population [166].

The reemergence is not just a public health concern for Nigeria only but has global health implication as trade in rodents have exported MPX to USA in 2003 [167,168] while human travelers from Nigeria have also exported the disease to Israel, Singapore, and the United Kingdom in 2018 and 2019, respectively [51,169,170,171]. More worrisome is that there is now evidence of human-to-human transmission of the West African clade of MPXV not just in Nigeria [60] but also in the United Kingdom [170]. Overall, MPX should no longer be considered a rare disease that is geographically limited to countries in the West and Central Africa. It is a global health threat, as well as a biodefence/biosecurity threat, and huge efforts must be directed in deciphering the zoonotic hosts, the epidemiological factors that maintain and sustain the virus in ecosystems, as well as the viral and host factors that modulate animal-to-human transmission and human-to-human transmission.

## 4. Phylogeny and Evolution of Monkeypox Virus in Nigeria

### 4.1. Phylogeny

**Figure 4:**
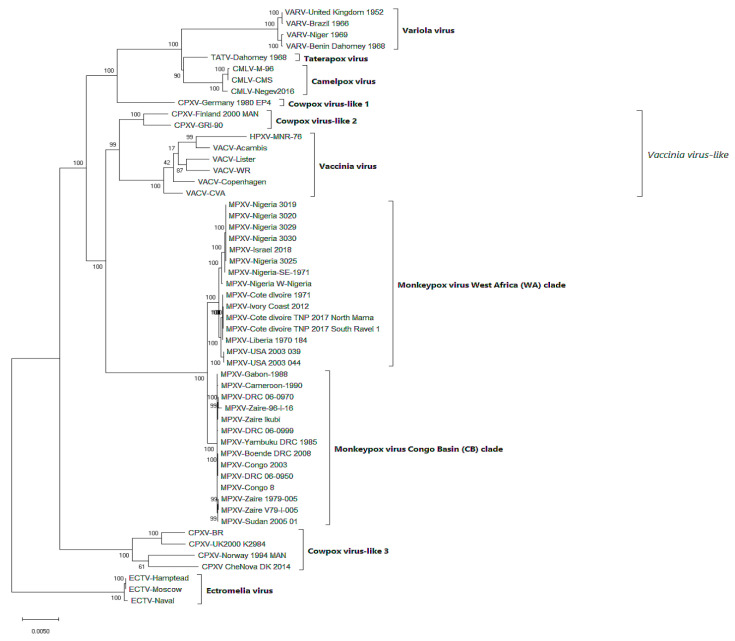
Maximum Likelihood (ML) phylogeny of orthopoxvirus genomes. Full-length genome sequences of 53 OPVs, including 23 MPXVs, were retrieved from The Virus Pathogen Database and Analysis Resource (ViPR) website [172]. The genomes were aligned with MAFFT version 7 [174]. The full-length genome alignment was stripped of gaps, and phylogenetic analysis was conducted, using Maximum Likelihood algorithm, as implemented in MEGA X [175]. Bootstrapping was set to 1000 replicates, and a General Time Reversal (GTR) model was used, as suggested by JModel test [181].

The genomic sequences of 29 MPXVs (eight Nigerian isolates including one export to Israel) and 23 other OPXVs, were retrieved from The Virus Pathogen Database and Analysis Resource (ViPR) website (https://www.viprbrc.org) [172]. Genomes of five Nigerian isolates (MPXV-3019, MPXV-3020, MPXV-3025, MPXV-3029, MPXV-3030) were from 2017 outbreak in Rivers State [60] and the two other genomes from Nigeria isolates (MPXV-SE-Nigeria, MPXV-W-Nigeria) were from earlier outbreaks in 1971 (Abia State) and 1978 (Oyo State), respectively [173]. The genome of the isolate exported to Israel (MPXV-Israel) in September 2018 was from an individual resident in PortHarcourt, Rivers state who returned to Israel a week after disposal of two rodent carcasses in his PortHarcourt residence [68]. All sequences were aligned by using MAFFT version 7 [174]. A phylogenetic tree was constructed from the aligned sequences, using a Maximum Likelihood (ML) algorithm as implemented in MEGA X software [175]. The tree shows that MPXVs were resolved into two major clades: the West Africa (WA) and the Congo Basin (CB) clades. The WA clade includes all the eight Nigerian isolates and other isolates from Liberia, the Ivory Coast, and the USA (exported from Ghana) (Figure 4). In addition, the WA clade has two major subgroups, the Nigerian isolates formed a subgroup that is distinct from a second sub-cluster consisting of isolates from Liberia, Ivory Coast and USA. MPXV-Nigeria W (isolated in 1978) and MPXV-Nigeria-SE-1971 are genetically distant from the other recent Nigerian MPXV-isolates (Figure 4). The monophyly of recent Nigerian isolates with the exported isolate to Israel suggest that the viruses may have emerged from the same infection pool. This is consistent with a recent Bayesian phylogenetic analysis of MPXVs exported from Nigeria to other parts of the world [176]. The CB clade consists of isolates from Democratic Republic of Congo, Sudan, Gabon, and Cameroon. Although Gabon and Cameroon are countries in West Africa, MPXVs isolated from these two countries resolved into the CB clade and these isolates may have originated from DRC since these Central African countries share the Congo Basin habitat. The MPXV tree topology in this paper is in agreement with previously published results [157,173,177]. Although genome-wide phylogenetic analyses of Nigerian MPXVs have been reported before [157], the results presented here represented the most comprehensive analysis, as it includes more genome sequences than previously documented. Moreover, the phylogenetic tree described here (Figure 4) is in conformity with what is generally known about OPV tree topology: (i) ECTV is the most distant species in the group [178], (ii) CPXV is polyphyletic, and CPXV-like 2 clade is more related to VACV clade than to other CPXVs [178,179]; (iii) VACVs are sister to MPXVs, and the resolution of VACV clade is clouded by low bootstrapping support [178,180]; and (iv) CMLV and TATV share a common ancestor, and are sisters to VARV [178,179]. Future studies should also look at the possibility of MPXV not having natural reservoir(s) but circulates in a wide variety of natural and incidental animal host species. Research studies on evolutionary divergence of the two clades should be done because it is possible that the two clades have two different reservoir species.

### 4.2. Recombination

Poxviruses undergo high-frequency recombination during infection of cells [182,183]. Among OPVs, naturally occurring inter-specie recombination events have been detected between CPXV and ECTV [182,184] and intra-species recombination between strains of VARV [185] and VACV [186]. However, natural recombination events have not been reported for MPXVs. Recombination is believed to be one of the drivers of poxvirus evolution [182], but Babkin did not find evidence in support of the role of recombination in OPV evolution [187]. Nevertheless, there is substantial evidence that tandem gene duplications are a result of recombination [188,189].

Estep et al. used homologous recombination to replace the D14L gene with an EGFP-GPT cassette in MPXV-Z genome in order to determine the role of MOPICE in MPXV pathogenesis [190], and a recombinant MPXV encoding green fluorescent protein was engineered in order to study MPXV infection in a monkey model [191]. The ease of construction of recombinant MPXVs in the lab raises the possibility that such recombination may occur or have occurred in nature between co-infecting MPXVs, MPXVs, and naturally occurring OPVs, including VARV, as well as smallpox vaccine strains. Genome-wide recombination analysis of Nigerian MPXVs isolated from humans and animals will shed light on the role of recombination, if any, on the evolution of MPXVs in Nigeria.

### 4.3. Gene Loss, Gene Gain, and SNPs

OPV evolution is also driven by variation in genome content. OPVs adapt to their host by gene loss and gain [192]. Genome-sequence length and gene content positively correlates with wide host range but has an inverse relationship with pathogenicity [193]. The WA clade of MPXV (in particular, the Nigerian isolates) have larger genomes (197,566–197,792 bp) and content compared to the CB clade (especially the Zaire isolates) (196,850–196,959 bp), and that may contribute to the less virulence of the WA clade [194]. This is underscored by studies in ground squirrels that demonstrated that animals infected with Congo Basin MPXV had more severe symptoms and died compared to West African MPXV challenged animals [35,94]. However, in some instances gene loss may result in attenuation instead of increased virulence as has been demonstrated with the evolution of Chorioallantois vaccinia virus Ankara (CVA) to Modified vaccinia virus Ankara (MVA) [194] or by the deletion of virulence genes in MPXV [91]. It follows that OPVs adopt reductive evolution by gene loss in order to optimize their response to the host antiviral mechanisms. There is a strong evidence that the main mechanism for gene loss is the introduction of early stop mutation (ESMs) that progressively lead to fragmentations, truncations, and complete deletion of the ORF [195]. Indeed, there is an inverse relationship between genome size and the combined total number of gene fragmentations, truncations, and missing ORF.

Recently, it was demonstrated that under selection pressure triggered by antiviral host response, OPVs deploy transient gene expansion to overcome the host antiviral assaults. Mutant VACV in which E3L was deleted was shown to counteract the antiviral activity of human protein kinase R (PKR) by increasing the copy number of K3L, expanding its genome size by 7–10% [196]. Tandem K3L duplication stopped once adaptive H47R mutation was acquired, and this was followed by genome contraction evidenced by the rapid loss of K3L copies without the H47R mutation [196]. It is likely that this novel but transient mechanism of gene expansion and homogenization of adaptive SNPs may also be drivers of evolutionary adaptation in other OPV species including MPXV. Two recent studies have used haploid network mapping based on SNP matrix of the whole genome to infer epidemiologically linked clusters for 2017 MPX outbreak in Nigeria [60] and exported MPX from Nigeria to the UK, Israel, and Singapore [176]. Both studies inferred that the PortHarcourt prison cluster (MPXV-3018, MPXV-3019, MPXV-3020, MPXV 2029) has an average SNPs (point mutation) of 1.5, whereas the average SNP for export related MPXV genomes were found to be 5.9 [176]. However, the problem with haplotype network mapping for MPXVs is that there is no demonstrated threshold above which epidemiological related cluster hypothesis can be rejected. For instance, there is variation in the average SNPs for known MPX epidemiological clusters; the 2003 USA MPXV and 2017 PortHarcourt outbreaks had average SNPs of 0.4 and 1.5, respectively. In addition, genomes from virus isolates from different outbreaks and geographical regions may have as few as three SNPs separating them (M5312 from Rivers vs. M2972 from Lagos), whereas samples from the same geographic region and outbreak has as much as 10 SNPs (MPXVs-3018/3019_Rivers vs. MPXV-3030_Rivers) between them [176]. Overall, haploid network mapping based on SNP matrices need to be corroborated by epidemiological, phylogenetic and phylogeographic evidences in order to identify epidemiological linked or unlinked clusters during MPX outbreaks in Nigeria and elsewhere.

## 5. Gaps in Knowledge, Omitted Research, and Conclusion

### 5.1. Reservoir Host Species and Tissue Tropism

There is no definitive reservoir or natural host for MPXV [60,136,197], but rodents and non-human primates have been determined to be potential natural reservoir and incidental hosts of the virus [198,199]. The difficulty in understanding the pathogen–host associations, the epidemiology, the natural history, and the ecological and climatic effects of MPXV could be ascribed to the detection of MPXV in a wide range of rodent species and non-human primates [74], as well as the paucity of data on natural reservoirs and maintenance hosts of the virus. Knowledge of tissue tropism will give a good grasp about the spread and the immune responses of the virus between the hosts [79]. There is no specific or definite tissue tropism of MPXV because it has been detected in a number of tissues, and this is no surprise as MPXV has been detected in a wide variety of animals. Ultimately, identifying the definite natural host of MPXV could lead to its specific tissue tropism. Future research should also focus in identifying the virus and host factors that modulate zoonotic spillover events, human-to-animal transmission, human-to-human transmission, and animal-to-animal transmission.

### 5.2. Co-Infection and Recombination

Recombination is one of the drivers of evolution among the *Poxviridae* family [195], and it is in part also responsible for most genetic and phenotypic diversity among OPVs [200]. However, little is known about recombination between MPXVs and other OPVs during co-infection or superinfection in a host, and the role of recombination in MPXV infection biology and pathophysiology. In Nigeria and other monkeypox endemic regions of West and Central Africa, it is not known whether or not MPXV co-infected or superinfected the same human host with VARV during the smallpox pandemic. Did potential natural superinfection of VARV in human hosts already infected with MPXV contribute to the eradication of smallpox in West Africa and was there attenuation of smallpox in West Africa due to cross-protection elicited by pre-existing MPXV antibodies? However, the low incidence of MPXV infection, low human-to-human transmission, and the observation that MPX is an acute rather than persistent infection makes superinfection or co-infection with VARV unlikely. While these speculations cannot be examined experimentally due to ethical consideration and dual use risk [201], analysis of historical/retrospective samples by using next generation sequencing and proteomic technologies [202,203] will shed light on the role of co-infection/superinfection, recombination and preexisting MPXV antibodies to VARV and MPXV infection biology. These studies are essential to our understanding of the host and viral factors that modulate MPXV pathogenesis and its possible emergence as a VARV-like pathogen. There are reports of co-infection of MPXV and VZV (Varicella-Zoster virus) [72,93,136,204,205,206] and co-infection of MPXV and HIV [206,207,208]. Ogoina et al. hypothesized that the alteration (in co-infected patients) in MPXV natural history and course of infection could be related to HIV immunosuppression. Thus, more studies are warranted to test this claim especially in Nigeria where MPXV and HIV co-infection has been reported [207].

### 5.3. Monkeypox Virus Infectome

Global analysis of virus and host gene expression patterns in MPXV infected cells has been done both in cell cultures and animal models [96,116,198,209,210]. Transcriptomic [209], proteomic [211], and system kinomics [96] all show the down regulation of host anti-viral responses especially in the CB clade compared to the WA clade [96,198,209]. Studies also demonstrated functional redundancy of some genes in MPXV since immune evasion strategies against the host antiviral arsenal were elicited although MPXV has truncated version of those genes [212,213]. While there are data on virus and host genes expression patterns, information is still scanty on the functional implication of these gene expression profiles to virus infection biology and the molecular basis of MPXV pathogenicity. Most published work on MPXV infectome was done with MPXV Zaire and MPXV WRAIR7-61 strains. While this may have provided insight into the inter-clade specific variability with regard to the virus infection biology, it supplied no answers with respect to intra-clade specific variability. Thus, there is no basis to assume that MPXV WRAIR7-61 infection biology will be similar to that of Nigerian isolates just because they belong to the same WA clade. In this connection further infectome studies should deploy more isolates from the two major clades. This will provide a more holistic picture of the infection biology of MPXV and possibly define specie-specific antiviral strategies.

### 5.4. Antibody-Dependent Enhancement (ADE) of Infection

Viral infection is reduced and neutralized by antiviral antibodies which are major component of the host immune response against viral infections, and ADE of infection occurs when these antibodies facilitate the replication and uptake of the virus into target cells and tissues. Kulkarni [214] hypothesized that antibodies elicited through vaccination may trigger ADE and increase the severity of the disease [214,215,216,217]. Although IgM and IgA antibodies alongside with their complement have penchant of ADE which has been observed in some viruses like Zika virus, Dengue virus and Ebola virus [214,217], none has been reported in MPXV cases. However, scenarios where humans, domesticated animals, and wildlife populations have pre-existing antibodies to MPXV or other naturally occurring OPVs and are then exposed to smallpox vaccine or natural superinfection with OPVs might conceivably enhance infectivity and change MPXV host range, tissue, and cell tropism Thus, examination of ADE in MPXV during natural and experimental infections will enhance our knowledge about the associated risk in using new generation smallpox vaccines, vaccine-induced antibodies, and antiviral immunoglobulins as therapy against MPX.

### 5.5. Conclusions

The wane in herd immunity as a result of cessation of smallpox vaccination, increased contact between humans and potential MPXV animal reservoir hosts as a result of climate change and deforestation, bush meat consumption, and inadequate health and research infrastructure among others may have created an immunological and ecological niche for MPXV to re-emerge in Nigeria and other countries within the tropical rain forest belt. MPX is no longer confined to the endemic regions as travelers have exported MPX from Nigeria and Ghana to the USA, the UK, Israel, and Singapore in recent years. Therefore, MPXV is a very serious re-emerging pathogen with global outreach. To prevent MPXV from occupying the ecological niche vacated by VARV and possibly evolve to a much deadlier pathogen than it is at present, national and global research efforts should be intensified in order to identify virulence markers of disease, host and viral factors that modulate MPXV evolution, human behaviors that support zoonotic spillover events, surrogates for asymptomatic infection, as well as virus and host determinants of immunity. In Nigeria in particular MPXV epidemiological surveillance in humans and potential host species should be pre-emptive, that is, it should be conducted on a routine basis and not just in response to an outbreak. Fennoscandia [218,219,220,221,222,223] and Germany’s [6,224,225,226] extensive surveillance for CPXVs, a cousin of MPXV, can serve as a model for MPXV surveillance in Nigeria. A recent deployment and implementation of the Surveillance Outbreak Response Management and Analysis System (SORMAS) for MPX outbreak in Nigeria by NCDC [147] is a step in the right direction, and we advocate that routine, periodic epidemiological surveillance for MPXV in humans and animals should be integrated into SORMAS.

## Figures and Tables

**Table 1:** Natural monkeypox virus (MPXV)-infected animals.

	Geographic Location/Countries	Method of Detection	References
Sooty mangabey monkey (*Cercocebus atys*)	Côte d’Ivoire	PCR	[66,84]
Gambian-pouched rat (*Cricetomys gambianus*)	Africa	PCR and viral isolation	[85,86,87,88,89]
Rhesus macaques (*Macaca mulatta*)	Copenhagen	Serological test	[15,90,91,92,93,94]
Cynomolgus Macaque (*Macaca fascicularis*)	Singapore/Copenhagen	Viral isolation	[49,92,95,96]
Asian Monkeys (*M. fascicularis*)	Copenhagen	Viral isolation	[97,98,99,100]
Southern opossum (*Didelphis marsupialis*)	South America	PCR and viral isolation	[88,89,98,99]
Sun squirrel (*Heliosciurus* sp.)	Zaire	Antibody detection test	[62,88,89,98,101,102]
African hedgehogs (*Atelerix* sp.)	Africa	PCR, antibody detection test, and viral isolation	[81]
Jerboas (*Jaculus* sp.)	Illinois, USA	PCR, antibody detection test, and viral isolation	[49,81]
Woodchucks (*Marmota monax*)	USA	PCR and viral isolation	[98,100]
Shot-tailed opossum (*Monodelphis domestica*)	USA	PCR and viral isolation	[98,100]
Porcupines (*Atherurus africanus*)	Zaire	PCR and viral isolation	[58,61,78,89]
Giant anteaters (*Myrmecophaga tridactyla*)	Rotterdam	Viral isolation	[103]
Prairie dogs (*Cynomys* spp.)	USA	PCR and viral isolation	[33,81,99,104,105,106]
Elephant shrew (*Petrodromus tetradactylus*)	DR Congo	Serological test	[99,107]
Domestic pig (*Sus scrofa*)	DR Congo	Serological test	[99,108]
Rope squirrel (*Funisciurus* sp.)	Zaire	PCR and viral isolation	[55,87,88,104,107,108,109]
African dormice (*Graphiurus* spp.)	USA	PCR and viral isolation	[55,104,109]
